# Supplementary material for: Systematic Evaluation of Cyanidioschyzon merolae Across Photobioreactor Systems: Linking Reactor Design to Biomass Production and Biochemical Composition
Source: Microb Biotechnol. 2026 Nov 2;19(11):e70468. doi: 10.1111/1751-7915.70468 (PMC13728316; doi:10.1111/1751-7915.70468)
Supplement: Supplementary file 1 [file MBT2-19-e70468-s001.docx]

**Supplemental information to:**

**Systematic evaluation of *Cyanidioschyzon merolae* across photobioreactor systems: Linking reactor design to biomass production and biochemical composition**

**Philipp Ernst^1,2,5^, Jan Vanselow^1,2^, Max Denter^1,2^, Wen Li^3^, Lennart Witting^1^, Jochem Gätgens^1^, Markus Pauly^4^, Dietrich Kohlheyer^1^, Vlada B. Urlacher^3^, Michael Feldbrügge^5*^ and Julia Frunzke^1,2*^**

^1^Forschungszentrum Jülich GmbH, Institute of Bio- and Geosciences, IBG-1: Biotechnology, Jülich, Germany

^2^Heinrich Heine University Düsseldorf, Faculty of Mathematical and Natural Sciences, Institute of Microbial Interactions, Düsseldorf, Germany

^3^Heinrich Heine University Düsseldorf, Faculty of Mathematical and Natural Sciences, Institute of Biochemistry, Düsseldorf, Germany

^4^Heinrich Heine University Düsseldorf, Faculty of Mathematical and Natural Sciences, Institute of Plant Cell Biology and Biotechnology, Düsseldorf, Germany

^5^Heinrich Heine University Düsseldorf, Faculty of Mathematical and Natural Sciences, Institute of Microbiology, Düsseldorf, Germany

*Corresponding authors: [j.frunzke@fz-juelich.de](mailto:j.frunzke@fz-juelich.de) and [feldbrue@uni-duesseldorf.de](mailto:feldbrue@uni-duesseldorf.de)

**Content:**

Figure S1: Calibration curves for relationship between optical density and cell dry weight of the three microalgae strains tested (n=2).

Figure S2: Growth rates at different light intensities and CO_2_ concentrations in the MC.

Figure S3: Raw cell count over time plots for individual replicates during microfluidic single-cell cultivation.

Figure S4: Purification of R-PC and R-APC from *C. merolae***.**

Figure S5: Fatty acids and elemental analysis of *C. merolae* endpoint samples.

Figure S6: Elemental analysis of *G. javensis* and *L. platensis* endpoint samples.

Video S1: Time-lapse image sequence displaying growth of *C. merolae* in a microfluidic cultivation chamber.

Video S2: Time-lapse image sequence displaying growth of *G. javensis* in a microfluidic cultivation chamber.

**Figure S1: Calibration curves for relationship between optical density and cell dry weight of the three microalgae strains tested (n=2).**

The linear equations were used to determine the cell dry weights from the optical density values of the cultivation samples.

**Figure S2: Growth rates at different light intensities and CO_2_ concentrations in the MC.**

*C. merolae* 10D day/night cycles (14/10 h). The mean values with standard deviation of two independent biological replicates are shown (n=2).

**Figure S3: Raw cell count over time plots for individual replicates during microfluidic single-cell cultivation.**

Growth rates were determined for both investigated strains by counting the number of cells within three separate image sequences. The growth rate (μ) was calculated by performing a linear fit on the ln-transformed cell count data. The mean of the individual replicates is displayed in Table 1.

**Figure S4: Purification of R-PC and R-APC from C. merolae.**

Chromatogram showing protein elution monitored at 280 nm alongside conductivity. After removal of unbound material, target proteins were eluted using a linear gradient of sodium chloride. PDB code: 1I7Y.

**Figure S5: Fatty acids and elemental analysis of C. merolae endpoint samples.**

Fatty acids (A) and elemental analysis (B) of the endpoint samples after cultivation of *C. merolae* in the MC (n=3).

**Figure S6: Elemental analysis of G. javensis and L. platensis endpoint samples.**

Elemental analysis of (A) *G. javensis* endpoint samples of IIPBR (n=2) and MC (n=3) and (B) *L. platensis* endpoints samples of FPR (n=1) and MC (n=3).

**Video S1: Time-lapse image sequence displaying growth of *C. merolae* in a microfluidic cultivation chamber (same as Figure 2A).** Each image shows an overlay of phase-contrast and fluorescence images (excitation: 514/30 nm; emission: 561 nm; emission: 629/56 nm) acquired during time-lapse microscopy. Images were taken every hour. During the cultivation, the chip was continuously illuminated with 30 µE white light. The temperature was maintained at 42 °C and the growth medium was perfused continuously at a rate of 200 nl∙min^-1^. The scale bar indicates a length of 2 µm.

*Video S1 is provided as supplementary file: Supplementary_video_S1_Cmerolae*

**Video S2: Time-lapse image sequence displaying growth of *G. javensis* in a microfluidic cultivation chamber (same as Figure 2B).** Each image shows an overlay of phase-contrast and fluorescence images (excitation: 514/30 nm; emission: 561 nm; emission: 629/56 nm) acquired during time-lapse microscopy. Images were taken every hour. During the cultivation, the chip was continuously illuminated with 30 µE white light. The temperature was maintained at 42 °C and the growth medium was perfused continuously at a rate of 200 nl∙min^-1^. The scale bar indicates a length of 2 µm.

*Video S2 is provided as supplementary file: Supplementary_video_S2_Gjavensis*
